# Recent advances in radiation oncology

**DOI:** 10.3332/ecancer.2017.785

**Published:** 2017-11-30

**Authors:** Cristina Garibaldi, Barbara Alicja Jereczek-Fossa, Giulia Marvaso, Samantha Dicuonzo, Damaris Patricia Rojas, Federica Cattani, Anna Starzyńska, Delia Ciardo, Alessia Surgo, Maria Cristina Leonardi, Rosalinda Ricotti

**Affiliations:** 1 Unit of Medical Physics, European Institute of Oncology, 20141 Milan, Italy; 2 Department of Radiation Oncology, European Institute of Oncology, 20141 Milan, Italy; 3 Department of Oncology and Hemato-Oncology, University of Milan, 20122 Milan, Italy; 4 Department of Oral Surgery, Medical University of Gdańsk, 80–211 Gdańsk, Poland

**Keywords:** image guided radiotherapy, adaptive radiotherapy, stereotactic body radiotherapy, intensity-modulated radiotherapy, radiogenomics

## Abstract

Radiotherapy (RT) is very much a technology-driven treatment modality in the management of cancer. RT techniques have changed significantly over the past few decades, thanks to improvements in engineering and computing. We aim to highlight the recent developments in radiation oncology, focusing on the technological and biological advances. We will present state-of-the-art treatment techniques, employing photon beams, such as intensity-modulated RT, volumetric-modulated arc therapy, stereotactic body RT and adaptive RT, which make possible a highly tailored dose distribution with maximum normal tissue sparing. We will analyse all the steps involved in the treatment: imaging, delineation of the tumour and organs at risk, treatment planning and finally image-guidance for accurate tumour localisation before and during treatment delivery. Particular attention will be given to the crucial role that imaging plays throughout the entire process. In the case of adaptive RT, the precise identification of target volumes as well as the monitoring of tumour response/modification during the course of treatment is mainly based on multimodality imaging that integrates morphological, functional and metabolic information. Moreover, real-time imaging of the tumour is essential in breathing adaptive techniques to compensate for tumour motion due to respiration.

Brief reference will be made to the recent spread of particle beam therapy, in particular to the use of protons, but also to the yet limited experience of using heavy particles such as carbon ions.

Finally, we will analyse the latest biological advances in tumour targeting. Indeed, the effectiveness of RT has been improved not only by technological developments but also through the integration of radiobiological knowledge to produce more efficient and personalised treatment strategies.

## Introduction

Radiotherapy (RT) plays a crucial role in the care of cancer with approximately 50% of all patients benefiting from RT in the management of their disease [1–3].

Radiation oncologists work with medical and surgical oncologists to coordinate a multidisciplinary approach to the management of cancer.

In the curative setting, RT can be offered as the sole radical treatment. It can also be combined with surgery, being given during (intra-operative), before (neoadjuvant) or after resection (adjuvant), or with systemic therapy, sometimes for organ preservation (such as larynx, breast, urinary bladder, etc) [4–6]. Moreover, it can provide symptom relief in cancers that are locally advanced or disseminated by reducing or eliminating pain from bone metastases in 60% of cases [7–9].

RT also has an effect on the dissemination of the tumour in that local/regional therapies are, in effect, ‘stopping metastases at their source’ [10]. More recently, the possibility of the abscopal effect has been raised on the basis of a remission in out-target lesions after localised RT [11].

RT is very much a technology-driven treatment modality. RT techniques have changed significantly over the past few decades due to the improvements in engineering and computing, evolving from conventional irradiation using simple treatment fields towards highly conformal RT techniques, such as intensity-modulated RT (IMRT), intensity-modulated arc therapy (IMAT) and stereotactic RT (SRT), which aim to improve the outcome by escalating the dose to the target and minimizing the toxicity to normal tissue and critical organs. So, nowadays, certain tumours (i.e. breast and prostate cancer) receive shorter courses of RT as a safe and well-tolerated alternative to the longer conventional schemes; this is a big advantage for patients and also for healthcare costs [12]. Indeed, high-precision extremely hypo-fractionated RT has been called virtual surgery, since in many situations it can have a radical curative effect locally that is similar to surgery. From the biological point of view, such a high dose per fraction induces different radiobiological mechanisms of cell killing and therefore introduces a new concept of radio ablation.

Technological advances have mainly been the result of integration of imaging information in every phase of the treatment, from simulation to planning to delivery. Indeed, treatment planning systems (TPS) provide sophisticated image registration and fusion algorithms [13]. Moreover, treatment planning optimisation is becoming more radiobiology-oriented, integrating local radiation damage models [14].

At present, the precise identification of target volumes for treatment planning is mainly based on the integration of radiological/metabolic imaging, such as magnetic resonance imaging (MRI) or positron emission tomography (PET), with computed tomography (CT) scan simulation [15–20]. Tumour localisation immediately before and during treatment delivery by means of image-guided techniques is becoming a part of clinical practice and is a fundamental prerequisite for high-precision RT [21–24]. As part of a comprehensive RT treatment process, adaptive RT (ART) techniques make it possible to modify the treatment plan during the course of RT in order to account for anatomical and biological changes [13, 25].

However, technological advances seem likely to reach a plateau in the near future. Indeed, if the tendency towards ever-more cost-effective treatments is to be continued, these must be linked to a better understanding of tumour biology. Although DNA damage is generally considered the primary event leading to radiation-induced cell lethality, numerous non-DNA-related mechanisms have recently been implicated in cellular response to radiation (i.e. the bystander effect and the radiation-induced signalling of epidermal growth factor receptor [26–28].

Many of the developments in understanding the effects of radiation are now leading to a new vision of targeted therapeutics, creating a challenge which we will probably be facing in the near future [29, 30].

In this article, we will review some of the advances that have led to modern high-precision RT as it is today, focusing on the discoveries that have been made in terms of new technology and cancer biology.

## Principle of radiotherapy

In RT, high-energy radiation is used to destroy cancer cells, by depositing energy that damages the genetic material of cells and blocks their ability to divide and proliferate further [31].

Photon beams, produced by linear accelerators (LINACs), are characterised by a high deposit of energy near to the body surface with an exponential decrease of energy release as a function of depth. One of the major limitations of photon RT is that the cells of solid tumours become deficient in oxygen. Tumour cells in a hypoxic environment may be as much as 2–3 times more resistant to radiation damage than those in a normal oxygen environment, and much research has been devoted to overcoming hypoxia [32].

In particle therapy, or hadrontherapy, radiation is propagated by travelling corpuscles such as protons or boron, carbon, neon ions [33] that have an antitumour effect independent of tumour oxygen supply because they act mostly via direct energy transfer that causes double-stranded DNA breaks. Compared to photons, charged particles have an inverted depth dose profile; that is, low incident energy deposition with a spike at the tail-end of its dose distribution (the Bragg peak) and essentially no dose beyond the end range. Consequently, hadrontherapy spares the uninvolved tissue distal to the target and generally deposits a lower dose to the tissue proximal to the target than do photons.

The goal of RT is to maximise the radiation dose to cancer cells while minimizing exposure to adjacent normal cells, hence achieving a high probability of local tumour control [tumour control probability (TCP)] with a low risk of normal tissue complications [normal tissue complication probability (NTCP)] [34]. RT is based on the idea that the DNA repair capacity is generally greater in healthy cells than in cancerous cells [35, 36].

Cell survival after RT is modelled by an exponential function that accounts for both direct, called alpha, and indirect, called beta, mechanisms of DNA damage. The alpha/beta ratio of these types of damage expresses the ability of the tissue to repair the damage. This repair ability is inversely proportional to the alpha/beta ratio. Historically, the RT schedules conceived exploited the difference in damage repair between tumour and normal tissue by delivering small doses of radiation over a prolonged period of time. The rationale for fractionated RT is based on the four Rs of RT: re-assortment, repair, re-oxygenation and repopulation [37], to which radiosensitivity was later added [38]. Tissue with a high alpha/beta ratio is less sensitive to a high dose per fraction or hypo-fractionated RT compared to tissue with a low alpha/beta ratio. Most tumours have a high alpha/beta ratio (about 10 Gy) and are therefore treated with the standard fractionation of 2 Gy/fraction, or with a hyper-fractionated regimen (dose/fraction < 2 Gy, usually given twice a day). In the past decade, experimental and clinical data have suggested that prostate and breast cancer may actually have a lower alpha/beta ratio than previously suspected [39–43]. This may be due not only to different tumour characteristics but also to cell variability within the tumour or to uncontrolled confounding factors, such as the presence of tumour hypoxia, repopulation or patient-to-patient variability [14].

Recent technological advances have made it possible to safely escalate the dose/fraction, which improves the therapeutic ratio of RT, that is, it increases the cure rate and reduces toxicity [43, 44].

## Technological advances

In the following, we will briefly present the high-precision RT techniques currently available in clinical practice together with the fundamental prerequisite to accurately localise the target volume during treatment planning and delivery. Finally, ART is introduced within the framework of personalised medicine.

### Treatment techniques: state of the art

#### Intensity-modulated radiotherapy

Intensity modulation [45] was introduced in the early 1990s as a further refinement in the delivery of three-dimensional conformal radiation therapy (3D-CRT). IMRT was made possible by use of computer-controlled multi-leaf collimators (MLCs) and advanced treatment planning optimisation algorithms that are able to create the desired dose variation inside the radiation field. As opposed to standard planning techniques, where the dose distribution can only be modified by means of a try and error approach (changing for instance the field weight, angle and shape), with IMRT, the radiation oncologist designates the doses and dose/volume constraints for the tumour and the surrounding normal organs and the TPS determines the optimal fluence of each field resulting in a tailored dose distribution (inverse planning).

In the past, IMRT was usually delivered using a conventional LINAC with a static field geometry. Developments in IMRT techniques have focused on reducing treatment times with arc therapy by converting multiple static field IMRT into continuously rotating gantry intensity modulation [46].

Examples of IMAT solutions include TomoTherapy Hi-Art (Accuray, Inc., Sunnyvale, CA, USA) [47], and volumetric-modulated arc therapy (RapidArc, Varian Medical Systems, Inc., Paolo Alto, CA, USA and VMAT, Elekta, AB, Stockholm, Sweden) [48].

**Figure 1.**
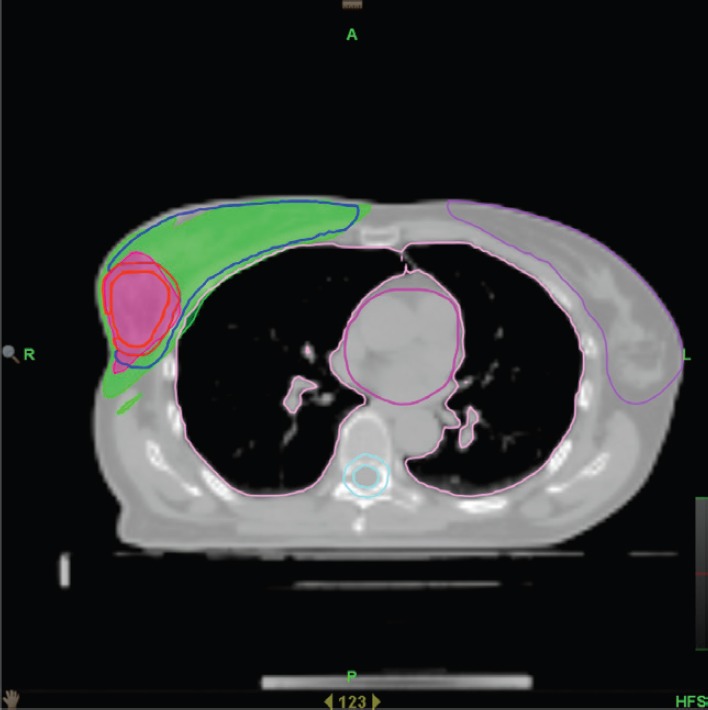
Axial view of a treatment plan for whole breast irradiation with SIB delivered with TomoTherapy. The colour green corresponds to 95% of the prescribed dose to the breast, and the colour red to 95% of the boost dose.

**Figure 2.**
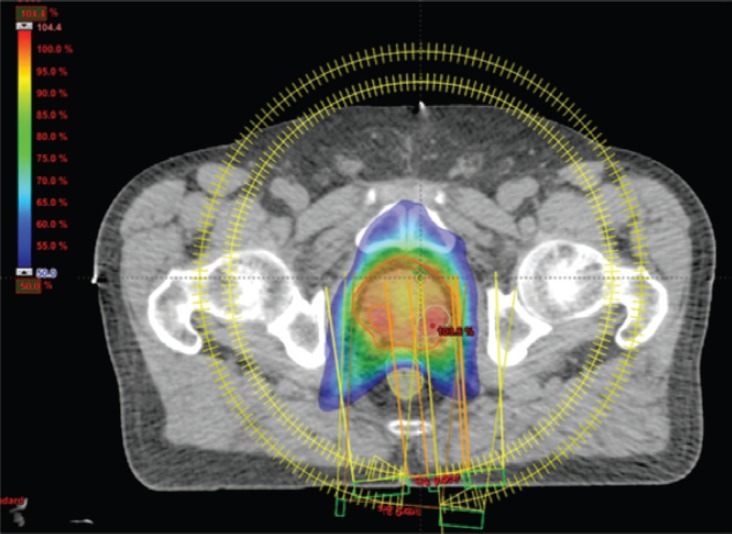
Axial view of a volumetric arc therapy (VMAT) treatment plan for prostate cancer delivered with RapidArc, with SIB to DILs.

IMRT/IMAT techniques make it possible to deliver different levels of dose to different parts of the tumour (for instance, a hypoxic area of the tumour, identified by means of functional imaging, may receive a boost dose). Approaches using a simultaneous integrated boost (SIB) and the delivery of dose-escalated conventionally fractionated or hypo-fractionated RT using IMRT techniques are now being investigated as an alternative to conventional RT for different anatomical sites: breast [49, 50], head and neck [51, 52] prostate [53, 54]. Examples, using tomotherapy, of SIBs delivered to the breast tumour bed and to the dominant intra-prostatic lesions (DILs) are shown in Figures 1 and 2.

However, with IMAT techniques, a larger volume of normal tissue receive a low radiation dose (dose bath) compared to static IMRT with a potential increase in induced second malignancies. Hall and Wuu [55] reported a theoretically increased risk of second malignancy from 1% to 1.75%. It is important to note that numerous epidemiological studies estimate that a large proportion of second cancers are related to other factors, such as lifestyle (smoking, alcohol, obesity, physical inactivity and diet), infections, (human papilloma virus, hepatitis C virus, etc) or genetics [56].

#### Stereotactic body radiotherapy

**Figure 3.**
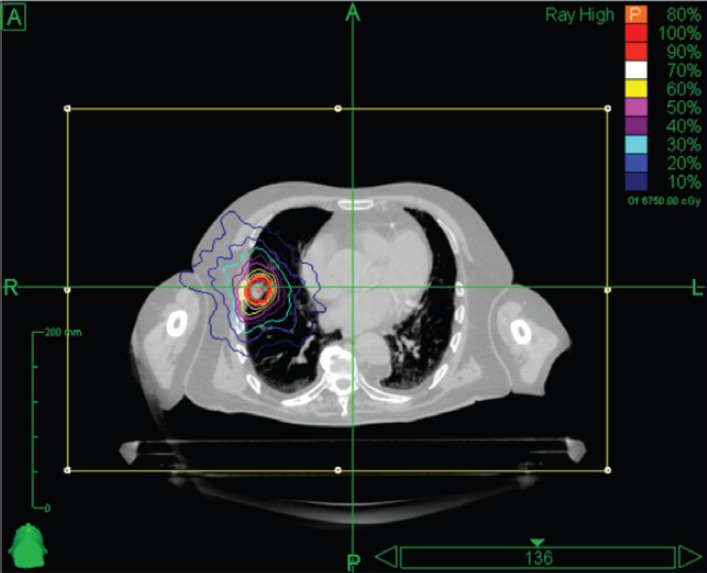
Axial view of a stereotactic treatment plan delivered with CyberKnife for an early stage non-small cell lung carcinoma.

Stereotactic body RT (SBRT) is very much a technology-driven treatment modality [57]. SBRT systems are capable of producing very conformal treatment plans with a steep dose gradient outside the target. This technique makes possible safe and efficacious treatment across a broad array of anatomic locations, in proximity to critical organs, and even adjacent to or within prior RT fields. Essential requirements for SBRT are the accuracy of target delineation (see ‘Tumour Localisation in Treatment Planning’ section), and the implementation of inter- and intra-fraction tumour motion compensation strategies (especially for tumours in the lung and in the upper abdomen). The wider availability of in-room imaging and advanced treatment delivery systems means that more institutions are now offering SBRT (see ‘Tumour Localisation in Treatment Delivery’ section) [58]. At present, there are a variety of systems available for SBRT. Some of them are based on the traditional LINAC gantry, for example, Versa HD (Elekta, AB, Stockholm, Sweden) [59] and TrueBeam STx (Varian Medical Systems, Inc., Paolo Alto, CA, USA) [60], whereas others have moved away from this design in search of greater non-coplanar beam arrangements, for example, CyberKnife® (Accuray, Inc., Sunnyvale, CA, USA) [61] and VERO (Mitsubishi Heavy Industries, Ltd., Japan, and BrainLAB AG, Feldkirchen, Germany) [62, 63]. An example of a stereotactic treatment plan delivered with CyberKnife® for early-stage non-small cell lung carcinoma is shown in Figure 3.

The local ablative capability of SBRT challenges surgery as the gold standard and could become the standard for patients with early stage lung cancer, who are operable but are at high risk of morbidity [64]. Indeed, SBRT has been called virtual surgery or radio ablation as in many cases, such as lung, it can have a radical curative effect locally that is similar to surgery.

#### Particle beam therapy

The past decade has seen an increasing use of particle therapy, particularly protons [65]. Radiation dose distributions for proton therapy often appear to be better than those for IMRT photon-based treatments, particularly in that they reduce the low and intermediate radiation dose to normal tissue. Proton therapy is new and although it has dosimetric advantages theoretically, independent evaluation is still to be made in order to assess its strengths and weaknesses [66, 67]. Indeed, prospective clinical trials to compare proton therapy to photon IMRT need to be carried out. Moreover, proton therapy is in the midst of a significant technological development at the level of motion management, evolving from passively scattered beams towards actively scanned ones [68–70]. Finally, proton therapy could be of use in a stereotactic regimen, but at the moment there is no clinical series that supports this hypothesis.

Proton therapy has been used internationally for cancers of the eye, base of skull and spine, particularly in paediatric patients [66, 71–73]. Indeed, proton therapy in children has been shown to have a lower incidence of vision and hearing impairment, of neurocognitive degeneration and of second cancers, than is the case with other RT modalities.

Moreover, heavy particles, such as carbon ions, are particularly indicated for severely radio-resistant tumours because their biological effectiveness is greater than that of photons and protons [74]. According to the Particle Therapy Co-Operative Group (PTCOG, www.ptcog.com), which constantly updates the statistics on cancer treatment with particle therapy, ten carbon ion therapy facilities are in operation to date (July 2017): five in Japan, two in China and five in Europe (Austria, Germany and Italy). The National Institute of Radiological Sciences Chiba, Japan, has been treating cancer with high-energy carbon ions since 1994, with more than 10,000 patients treated by August 2016 and, thus, is the centre with the greatest experience in carbon ion treatment worldwide [75, 76]. For the first time, at the National Centre for Oncological Hadrontherapy in Pavia, Italy, carbon ions delivered with active scanning together with breathing synchronisation and rescanning modalities have been used to treat patients with tumours of the liver and pancreas [77].

### Advances in treatment planning

The advent of sophisticated treatment delivery techniques has made it necessary to develop advanced TPSs.

Techniques that can integrate images acquired at different times is becoming a part of common clinical practice for accurate tumour localisation, 4D treatment planning and ART (see ‘Tumour Localisation in Treatment Planning’ and ‘Tumour Localisation in Treatment Delivery’ sections). Image registration and fusion, that is, the ability to identify corresponding spatial locations in two or more image volumes and to visualise the result by superimposing the images, is essential in a modern TPS [13]. A crucial aspect, when using registration techniques, is the accuracy of image realignment. Rigid registration algorithms perform translations, rotations and affine image transformations and are widely used in the clinical setting. However, since the human body is intrinsically deformable, rigid techniques often provide insufficient registration accuracy. Thus, elastic or deformable methods are required to cope with local differences between images [78]. These methods are capable of warping the target image locally to align it with the reference image. Non-rigid image registration is a challenging issue as a large number of parameters describing the spatial correspondence of images are needed. Most TPSs now support both rigid and deformable image registration and fusion [79].

The advances in technology have led to an escalation of the prescription dose or a change in the number of fractions. In recent years, increased attention has been paid to the radiobiological optimisation of the treatment plan, using TCP and NTCP models [14]. Even though dose–volume optimisation techniques are a mainstay of current TPSs, the biological optimisation used in IMRT planning is able to reduce radiation-induced toxicity [80–91].

In addition, although advanced inverse-planning techniques have led to the development of more automated approaches, plan optimisation is still a very time-consuming task with output varying greatly according to the experience of the operator. At the moment, the move towards using fully automated TPSs clinically is slow and debates are ongoing [84]. The arguments against automated treatment planning have concentrated on the examples of those treatments that are difficult to do automatically, such as bilateral post-implant chest wall irradiation.

### Tumour localisation in treatment planning

As mentioned earlier, the more precise radiation delivery becomes, the more important it is to accurately identify the extension of both the tumour mass and also the normal tissue and critical organs involved in the neoplastic degeneration. This is essential in order to optimise irradiation geometry by delivering the radiation dose to the tumour itself while minimizing the dose delivered to surrounding tissue and organs at risk (OARs).

The integration of radiological/metabolic imaging, such as MRI and PET, with the CT scan simulation can provide useful information for accurately visualizing the tumour volume [16, 17]. The integration of these images is made possible by image registration algorithms incorporated into the TPS. Technological developments mean that MRI has become increasingly useful in identifying and characterizing lesions within the prostate as well as detecting local disease recurrence following primary definitive prostate treatment. The inclusion of multiple MRI parameters is known as multiparametric MRI (mpMRI). Overlapping modalities in mpMRI corrects for deficiencies inherent in any individual sequence. Combining advanced techniques of functional MRI, including T2 weighted (T2W), dynamic contrast enhanced and diffusion weighted imaging (DWI), improves visualisation and the accurate detection of intra-prostatic lesions and differentiates between low and intermediate/high-grade disease [18, 44]. PET with different tracers has made it possible to obtain metabolic information and identify the most radio-resistant sub-volumes within the tumour [19, 20]. Adequate contouring is the fundamental pre-requisite for an effective and safe treatment plan. However, it is a process prone to errors and inter- and intra-operator variability [85, 86]. The delineation of tumour volumes is based on a complex process of data interpretation (clinical history, pathology and imaging). To improve the consistency of contouring among radiation oncologists, several working groups have provided consensus instructions and atlases [87–91]. However, it should be noted that no one guideline can be adopted as the perfect recipe for all patients. These protocols are often derived from ‘expert opinion’ and consensus as well as objective evidence, so it is not surprising that there can be variation between groups. Besides, the possibility of microscopic spread of the tumour beyond the anatomic borders defined by the atlases should be carefully considered by radiation oncologists, especially when high-precision techniques are used.

Generally, with the introduction of IMRT techniques into the clinical scenario, a large number of OARs is delineated to assess the low-dose bath. A recent development in RT is the use of automated atlas-based auto-segmentation algorithms to support contouring OARs [92, 93]. Several studies have demonstrated that inter-observer variability decreases when the automatic atlas-based segmented structures are used as a base and then modified [94–97]. Automatic or semi-automatic (needing manual revision) segmentation algorithms can speed up the delineation of OARs and they offer reliability and repeatability in delineating the structures.

### Tumour localisation in treatment delivery

The increasingly conformal dose distributions that are possible with modern RT make it even more important that the patient’s position is the same for the simulation and for the treatment. The sensitivity to treatment uncertainties due to organ motion and inaccurate patient positioning is even more worrying when high-precision techniques are combined with dose escalation and hypo-fractionation schemes.

#### Image-guided radiotherapy

Technological innovations have made possible the direct integration of imaging technology into the radiation treatment device to increase the precision and accuracy of radiation delivery by controlling the placement of the dose within the body [22, 98].

A broad range of image-guided radiotherapy (IGRT) modalities is now available and generally used. There are several methods for localizing the target for each treatment fraction: by localizing surrogates, including implanted fiducial markers, external surface markers or anatomical features (through planar imaging, fluoroscopy, kilovoltage CT (kV-CT) or megavoltage CT (MV-CT), MRI, ultrasound and x-ray imaging, electromagnetic localisation, optical surface imaging and so on. Depending on the imaging methods used, the IGRT systems may broadly be divided into radiation based, non-radiation based and hybrid systems [21, 99, 100].

Of all soft-tissue based IGRT techniques, cone beam CT (CBCT) is the most widely used. It consists of acquiring multiple projection radiographs (for head and neck imaging ~350, for thoracic/pelvic imaging up to 600) before the RT fraction and within a gantry rotation of 180°–360°. A volumetric image with high spatial resolution and sufficient soft-tissue contrast is reconstructed and registered to the reference planning CT to determine the correct target position. Translational and rotational positioning errors can be corrected online before irradiation [23, 24, 101–103].

To mitigate the effects of tumour motion due to respiration on image quality and registration uncertainty, CBCT can be acquired in conjunction with breath-hold strategies [104] or in a respiratory triggered approach (4D-CBCT) [105]. Moreover, ultrafast ‘snapshot’ volume imaging is ready to be deployed clinically [106].

Surface imaging devices, either based on passive infrared markers or active markers, can be used as a complementary positioning device, providing intra-fraction patient surveillance which further improves the overall precision of the treatment [107–114].

IGRT has been improved by the development of devices making it possible to reposition the patient using MRI. MRI yields superb soft-tissue visualisation and provides several imaging modalities for identifying movement, function and physiology without delivering any additional dose to the patient. Various integrated MRI-guided radiation therapy systems have been developed, such as the hybrid MRI–LINAC [115, 116], which is still a prototype, and the Viewray system (MRIdian System, ViewRay, Inc., Cleveland Oakwood Village, OH, USA) [117], which has been in clinical use for the last couple of years.

IGRT not only makes it possible to improve the accuracy of the treatment by minimizing the inter-fractional position uncertainties, but it is also able to monitor systematic changes in the shape and position of tumour volume and of normal tissue (weight loss and tumour regression), which means that the plan can be suitably modified.

#### Breathing adaptive radiotherapy

Real-time monitoring of patient position significantly reduces intra-fraction movement, due either to physiological movement as in the case of the prostate, or due to respiration when tumours are located in the lung or upper abdomen. Electromagnetic technologies such as implanted radiofrequency markers have been successfully used for the prostate [118, 119]. Marker-based real-time image guidance has been in clinical use in the CyberKnife systems for over a decade. For its use to become widespread, real-time IGRT will probably need markerless solutions. A variety of kilovolt-based and MV-based possibilities have been proposed [120]. Cine MRI, which is available with the new MRI-guided radiation therapy systems, is able to provide non-invasive target localisation during RT treatment [121, 122].

The American Association of Physicists in Medicine Task group 76 [123] recommends caution when using IMRT techniques in regions where respiratory motion may jeopardise treatment. At least two intra-fraction motion effects may be present in the intensity-modulated treatment of a target subject to respiratory-induced motion: the dose blurring and the interplay effect. Planning studies show that during IMRT treatments a cyclic breathing motion with a 5–30-mm amplitude has a significant effect on dosimetry [124–126].

Several techniques have been proposed to reduce the effects of respiratory motion, from simple motion-encompassing, respiratory gating, either in free-breathing or in breath hold, to dynamic tumour tracking (DTT) [116, 127]. With the motion-encompassing technique, the mean position and the range of tumour motion are estimated from fluoroscopy or four-dimensional CT (4D-CT) data to define the internal target volume (ITV). However, a large ITV is required to accommodate tumour motion, which may lead to an increase in normal tissue complications. Respiratory gating, in free-breathing or in breath-hold, reduces the size of the ITV, but it increases the overall treatment time since the irradiation is delivered only in a predefined phase of the respiratory cycle, usually end expiration or end inspiration (in the case of deep inspiration breath hold), and it might be uncomfortable for the patient [127]. However, deep inspiration breath hold reduces the dose to the heart and left lung in the case of irradiation to the left breast [128]. The most sophisticated and efficient technique to reduce the size of the ITV is DTT, which makes it possible to reposition the beam dynamically according to the position of the tumour once the real-time IGRT system has determined tumour location. This can be done by repositioning the radiation beam to the target using a dynamic MLC [128], a robotic LINAC (CyberKnife) [130] or a linac mounted on two orthogonal gimbals allowing pan and tilt rotation of the beam, VERO [113, 131]. Another method, which has been discussed in several studies but has yet to be clinically implemented, consists of repositioning the patient to the beam by means of a dynamic couch [132]. A recently published comparison of the four tracking technologies showed that all systems are capable of highly accurate target delivery in the presence of motion, and that large treatment errors resulted when motion was not explicitly accounted for [133].

#### Adaptive radiotherapy

ART and dose painting strategies are the most recent tailored treatments.

ART is a closed-loop radiation treatment process where the treatment plan can be adjusted using measurement feedback. The term ART usually refers to: 1) changing the treatment plan during a course of RT to account for temporal changes in anatomy (e.g. tumour shrinkage, weight loss, internal motion or change of OARs), 2) adjustment of the delivered dose based on early tumour response and 3) adaptation of the treatment strategy based on early response (e.g. adding chemotherapy or hypoxic sensitisers).

ART is very much dependent on the anatomical information provided by IGRT and relies on deformable image registration algorithms now incorporated in several TPSs [13]. Image-guidance cannot fully correct for non-rigid changes and, in general, a single plan produced before treatment is not sufficient to describe the actual delivered doses and often leads to suboptimal treatment. Treatment delivery can be changed daily or weekly to compensate for changes in patient anatomy (e.g. tumour shrinkage, weight loss or internal motion) as seen on the images acquired daily at the treatment unit. Various techniques have been put forward, from the simple off-line strategies based on a limited number of imaging data to the more sophisticated ‘plan of the day’ approach which makes it possible to compensate for day-by-day variations in the target volume and position.

The distribution of the delivered dose can be adapted to changes in tumour biology/function (e.g. hypoxia) as measured by functional imaging acquired during the course of treatment [25].

This technique requires advanced TPSs that are able to perform deformable image registration to take into account the changes in the target volume and surrounding OARs and the dose accumulation algorithm. The latter makes it possible to calculate the dose distribution on each imaging set (CT or CBCT) and then to calculate the delivered dose distribution as the accumulation of the single treatment plans.

A more appealing approach is the integration of molecular imaging into the anatomical information with the aim of identifying radiation-resistant regions within the tumour, such as high clonogen density, proliferation or hypoxia, as different tumour regions have a different radiosensitivity, which may make a heterogeneous dose distribution desirable in order to obtain greater tumour control.

There are two methods of achieving dose painting to biologic image-defined regions within a target: dose painting by contours and dose painting by numbers [134, 135]. The first prescribes the dose within biological image-defined contours of the target, while the second prescribes dose to voxels throughout the target as a function of signal intensity of the corresponding voxel in a biologic image. One critical issue is the ability to accurately visualise in space and time the exact location of those areas expressing a phenotype that may require the delivery of a higher radiation dose. This raises not only the availability of specific tracers for the various biologic pathways of interest but also the spatial resolution of the available imaging modalities. Furthermore, the dynamics of tumour biology with its temporal and spatial changes have to be taken into account.

The implementation of ART schemes presents several challenges. It requires the acquisition of repeated anatomical and/or functional images during treatment and the use of deformable image registration algorithms for target volume propagation and plan summation. It is still therefore rarely used in clinical practice because it is so time-consuming.

## Biological advances in tumour targeting

RT is moving towards the era of personalised medicine and away from the wrong assumption that ‘one size fits all’ and is offering individualised treatment strategies. The focus on individual variability will lead to a paradigm shift from common population based medicine to personalised and participative medicine [136, 137].

As we know, the efficacy of RT is limited by the intrinsic radioresistance of tumour cells, which means an increased risk of local tumour recurrence, so the need to overcome radioresistance and improve radiosensitivity explains why there is such great interest in identifying new molecules that have a synergistic effect with radiation.

One way to enhance the efficacy of RT that is already in use is to give chemotherapy or targeted agents concomitantly in order to modify the radiosensitivity of the tumour cells at the molecular level [138]. This field of radiation and cancer biology is rapidly expanding to provide a selective improvement in the tumour response to radiation, including T-cell checkpoint inhibitors, hypoxic radiosensitisers and cytotoxins, antiangiogenic agents, DNA repair inhibitors, signal transduction blockers, chemokine inhibitors and oxygen metabolism modifiers. Thus, there is a huge gap between the many exciting ideas emerging from pre-clinical studies in modern radiation and tumour biology and the lack of clinical trials testing these new concepts [139].

Furthermore, the immunotherapy field offers exciting prospects, with a drastic increase in immunotherapeutic protocols within the standard anticancer regimens. Similar progress is being made in radiobiological knowledge, as reported in a recently published review on the relation between RT and immunotherapy. What appears clear is that: 1) RT not only has a direct cytotoxic effect on tumour cells, but also reprograms the tumour microenvironment to exert a potent antitumour immune response, 2) RT enhances antitumour immunity, but also induces immunosuppressive responses and 3) the combination of immunotherapy and RT presents a multimodal treatment approach that involves stimulating and suppressing various pathways [140].

While numerous pre-clinical studies on RT-immunotherapy combination regimens have been reported, it is obviously necessary to carry out clinical and translational studies to explore what are the optimal RT doses, fractionation, timing and sequencing, and how they interact with various kinds of immunotherapy. Moreover, therapeutic success depends on the careful selection of immunotherapy agents and suitable patients [141].

At the same time, attempts are being made to identify the factors which might help to predict patients’ response to RT treatment. Predicting the response to RT by distinguishing between radioresistant and radiosensitive patients could be useful in minimising the risk of unnecessary treatment and the related side effects. Identifying biomarkers that can predict the sensitivity or resistance of tumours to radiation therapy and the risk of developing toxicity is another promising area of research [142].

Several investigators are attempting to apply the field of ‘omics’ to tailor individual treatment in order to obtain a better outcome in cancer therapy by means of the expression of genes, proteins and metabolites [143]. In radiation oncology, ‘omics’ may be able to predict the treatment response by screening for genetic polymorphism or by gene expression analysis, and assessing the potential of epigenetic factors, posttranslational modification, signal transduction and metabolism. An example in the plethora of ‘omics studies’ was published recently: a patient-specific molecular signature of radiation sensitivity used to identify the optimum RT dose; a gene expression-based radiation-sensitivity index and the linear quadratic model to derive the genomic-adjusted radiation dose (GARD) [144].

Besides the above mentioned ‘radiogenomics’, another promising area of ongoing research is ‘radiomics’ which aims to identify non-invasive imaging biomarkers. Radiomics analyses numerous medical imaging features, to which are added critical and interchangeable information regarding tumour phenotype [145]. In addition, radiomic features provide a complete and full representation of the entire tumour and capture intra-tumour heterogeneity. Radiomic features have been shown to be related to tumour histology, tumour grade/stage, patient survival, metabolism and other clinical features. Furthermore, some radiogenomic studies have reported an association between radiomic features and the underlying gene expression patterns [146].

It is thought that intra-tumour heterogeneity has potentially profound implications for clinical prediction (i.e. treatment response, survival outcomes, disease progression, etc) and consequently it is deemed to be an essential element of precision oncology.

## Conclusion

In conclusion, RT has undergone tremendous progress over the years, realising technological developments that have revolutionised its clinical use, but we must not forget the multifaceted nature of this discipline that makes it an interface between physics, chemistry, biology and medicine [147]. Only by exploring all these aspects will we manage to produce individualised radiation therapy with better target delineation, avoidance of normal tissue, dose escalation, dose fractionation and better prediction of treatment response.

## Author contributions

Cristina Garibaldi and Barbara Alicja Jereczek-Fossa equally contributed to the work and should be considered as first authors.
